# Number of ICD-10 diagnosis fields required to capture sepsis in administrative data and truncation bias: A nationwide prospective registry study

**DOI:** 10.1371/journal.pone.0320054

**Published:** 2025-03-19

**Authors:** Nina Vibeche Skei, Jan Kristian Damås, Lise Tuset Gustad

**Affiliations:** 1 Department of Intensive Care and Anesthesia, Nord-Trondelag Hospital Trust, Levanger, Norway; 2 The Mid-Norway Centre for Sepsis Research, Institute of Circulation and Medical Imaging, Norwegian University of Science and Technology (NTNU), Trondheim, Norway; 3 Centre of Molecular Inflammation Research, Institute for Clinical and Molecular Medicine, Norwegian University of Science and Technology (NTNU), Trondheim, Norway; 4 Department of Infectious Diseases, St. Olav’s University Hospital, Trondheim, Norway; 5 Faculty of Nursing and Health Sciences, Nord University, Levanger, Norway; 6 Department of Medicine and Rehabilitation, Levanger Hospital, Nord-Trondelag Hospital Trust, Levanger, Norway; Christus Oschner St. Patrick Hospital, UNITED STATES OF AMERICA

## Abstract

**Background:**

In observational studies that use administrative data, it is essential to report technical details such as the number of International Classification of Disease (ICD) coding fields extracted. This information is crucial for ensuring comparability between studies and for avoiding truncation bias in estimates, particularly for complex conditions like sepsis. Specific sepsis codes (explicit sepsis) are suggested to be identified by extracting 15 diagnosis fields, while for implicit sepsis, which comprises an infection code combined with acute organ failure, the number of diagnosis field remains unknown.

**Objective:**

The objective was to explore the necessary number of diagnosis fields to capture explicit and implicit sepsis.

**Materials and methods:**

We conducted a study utilizing The Norwegian Patient Register (NPR), which encompasses all medical ICD-10 codes from specialized health services in Norway. Data were extracted for all adult patients with hospital discharges registered with explicit and implicit sepsis codes from all Norwegian hospitals between 2008 through 2021.

**Results:**

Out of 317,705 sepsis admissions, we identified 105,499 ICD-10 codes for explicit sepsis, while implicit sepsis was identified through 270,346 codes for infection in combination with 240,789 codes for acute organ failure. Through our analysis, we found that 55%, 37%, and 10% of the explicit, infection, and acute organ failure codes, respectively, were documented as the main diagnosis. The proportion of explicit and infection codes peaked in the primary diagnosis field, while for acute organ failure codes, this was true in the third secondary diagnosis field. Notably, the cumulative proportion reached 99% in diagnosis field 10 for explicit codes and in diagnosis field 13 for implicit codes.

**Conclusion:**

Expanding the utilization of multiple diagnosis fields can enhance the comparability of data in epidemiological studies, both internationally and within countries. To make truncation bias visible, reporting guidelines should specify the number of diagnosis fields when extracting ICD-10 codes.

## Introduction

International Classification of Disease (ICD) codes are used to describe patients clinical characteristics and outcomes in hospital records, and these are often abstracted for research purposes [1]. The number of ICD-codes needed to capture an event have been the focus of the World Health Organization since 1967 [2]. As sepsis is a complex and heterogeneous syndrome defined as a life-threatening organ dysfunction caused by a dysregulated response to infection [3], the number of codes needed to capture sepsis has included both specific sepsis codes (explicit sepsis) and implicit sepsis codes [4,5]. The latter consists of a combination of two codes, i.e., a code for infection and a code for acute organ failure. In such complex clinical problems, one American study showed that the number of secondary ICD diagnosis fields extracted should probably be 15 or more to avoid that relevant ICD-10 codes are truncated [6], and otherwise introducing truncation bias. Truncation bias can lead to underestimation of incidence, gaps in the medical knowledge base, hindering efforts to improve patients outcomes, hampered political decision-making and decreased comparability of studies [7].

The number of diagnoses needed to capture sepsis in administrative data has changed with evolving definitions. The Angus definition (2001) included 1286 codes for infection and 13 codes for acute organ dysfunction, which Rudd et al expanded in 2020 [4,5]. How many diagnosis fields that are required to capture all these combinations are relatively unknown. One study found that catheter-related bloodstream infection and postoperative sepsis showed the greatest susceptibility to truncation bias, with high proportions of relevant ICD- codes appearing in the sixth secondary diagnosis field or beyond [6]. Recommendations for how many secondary diagnosis fields that are needed to capture implicit in order to avoid truncation bias are sparse. We thus previously used up to twenty diagnosis fields when describing trends in sepsis hospitalizations, in-hospital mortality and beyond [8]. As other studies report which diagnoses they use, but not how many diagnosis fields are used to capture these, it was difficult to directly compare the results with other studies.

Therefore, to inform future sepsis researchers and increase comparability, the objective of this study was to describe the number and percentages of ICD sepsis diagnosis codes found per diagnosis fields one through twenty for explicit and implicit sepsis. The secondary objective was to describe the cumulative percentage of sepsis diagnosis captured by increasing diagnosis fields one through twenty for explicit and implicit sepsis.

## Materials and methods

### Data source

We conducted a descriptive registry study using the population-based Norwegian Patient Register (NPR). The NPR includes data from all Norwegian specialized healthcare services, including hospitals, outpatient clinics or contract specialists. Since 2008, it has been mandatory to report individual diagnostic data using the ICD-10 codes, ensuring a complete national data set [9]. Previous studies have demonstrated a comprehensive coverage of ICD-10 data in the NPR [10,11]. The NPR allows for an unlimited number of diagnoses [9]. However, due privacy considerations, we extracted ICD-10 codes from the primary diagnosis, co-existing primary diagnosis and up to 19 secondary diagnosis fields. Pertinent variables provided by the NPR include demographic information, ICD-10 diagnostic codes, treatment codes, and dates of service. This comprehensive data set allows for detailed analysis of healthcare utilization and outcomes across the Norwegian population [9].

Sepsis was identified and classified using ICD-10 codes based on the Sepsis-3 definition [3]. Between January 1, 2008, and December 31, 2021, data were extracted individually for patients over 18 years old from all Norwegian hospitals. This was done using ICD-10 codes for infection combined with acute organ failure (implicit sepsis) and specific sepsis codes (explicit sepsis). Clinical sepsis codes (R-codes; e.g., R57.2 septic shock) are only valid in national guidelines in combination with other codes (e.g., infection code) [11], thus the R-codes were included in acute organ failure category. Infection, acute organ failure, and explicit sepsis codes were classified as binary variables (0 and 1), i.e., either absent or present. These codes were retrieved from the primary diagnosis fields, co-existing primary diagnosis field, and 19 secondary diagnosis fields. As the percentage of ICD-10 codes in the co-existing primary diagnosis field to the primary diagnosis was only 0.1% in each category, we merged the co-existing primary diagnosis field with the primary diagnosis and named the diagnosis field 1. The secondary diagnosis fields were denoted as diagnosis field 2 through 20. Details on the ICD-10 codes extracted are previously published [12].

### Statistical analysis

Descriptive statistics were employed to analyze data, and results are presented as numbers and frequencies with percentages, means with standard deviations and medians as appropriate.

Demographic characteristics of interest included sex, age, and age group (18-29, 30-39, 40-49, 50-59, 60-59, 70-79, and above 80). Clinical variables included explicit, implicit and acute organ failure codes. A Chi-square test of independence was employed to assess the association between explicit and implicit sepsis on the variables of sex, age and age group. This test was chosen to determine if there were statistically significant differences in the distribution of categorical responses between the two groups. Statistical significance was set at a p-value of < 0.05.

For every diagnosis field, we counted the number of explicit sepsis, infection, and acute organ failure ICD-10 codes and calculated the proportion by dividing the number of each code by the total number of corresponding diagnoses. We then reported the proportion of codes per group (explicit, infection, or acute organ failure) for each diagnosis field, as well as the cumulative proportion. We used the Stata software package (version 16, StataCorp, TX, USA) for all statistical analyses.

### Ethics

The study was approved by the Regional Committee for Medical and Health Research Ethics (REK) in Eastern Norway (2019/42772) and the Data Access Committee in Nord-Trondelag Hospital Trust (2021/184). In accordance with the approval from REK and the Norwegian Health Research Act, obtaining written consent from patients was not required for our project. The data were de-identified by NPR using specific serial numbers, ensuring that the authors could not identify individual participants. The analyses were conducted in the Services for Sensitive Data at the University of Oslo.

## Results

**Fig 1:**
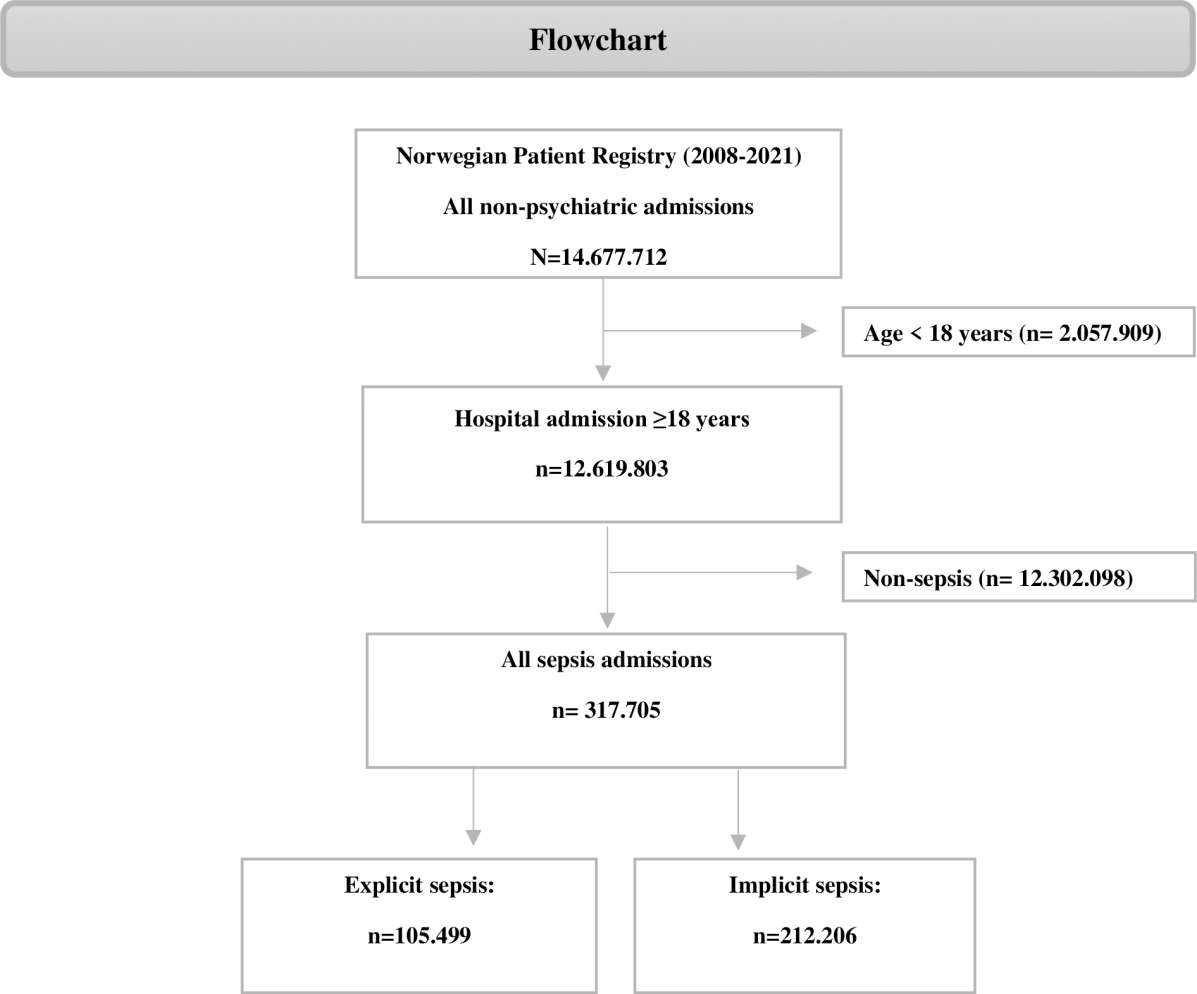
Flowchart of exclusion and inclusion process.

Out of 12.6 million discharges between 2008 and 2021, 317,705 discharges had one or more ICD-10 sepsis codes. Of these, 105,499 (33%) ICD-10 codes were identified for explicit sepsis, while implicit sepsis was identified in 212,206 (67%) admissions (Fig 1).

Men were over-represented in admissions with explicit (56%) and implicit sepsis (54%) (Table 1).

**Table 1:** Characteristics of the study population (2008-2021).

Variables	Total sepsis admissions^***^ n (%)	Explicit sepsis^*^ n (%)	Implicit sepsis^**^ n (%)	p-value^****^
**Hospital admissions**	**3** **1** **7 705 (100)**	**105 499 (33)**	**212 206 (67)**	
**Sex**				
Male	173 934 (55)	59 817 (56)	114 117 (54)	p < 0.001
Female	143 771 (45)	45 682 (44)	98 089 (46)	p < 0.001
**Age**				
Mean ± SD (Median)	71.0 ± 16.2 (73.9)	68.1 ± 17.3 (71.0)	72.5 ± 15.4 (75.1)	p = 0.14
**Age group**				p < 0.001
18-29	9 109 (3)	4 371 (4)	4 792 (2)	
30-39	9 665 (3)	4 514 (4)	5 151 (2)	
40-49	15 984 (5)	7 213 (7)	8 771 (4)	
50-59	30 792 (10)	12 292 (12)	18 500 (9)	
60-69	61 321 (19)	21 972 (21)	39 349 (19)	
70-79	84 830 (27)	25 357 (24)	59 473 (28)	
80 ^+^	106 004 (33)	29 834 (28)	76 170 (36)	

* Explicit sepsis includes explicit ICD-10 sepsis codes, regardless of implicit ICD-10 sepsis codes or not at the same admission

** Implicit sepsis includes only implicit ICD-10 sepsis codes at the same admission, excluding explicit sepsis admissions

*** Total sepsis admissions =  explicit sepsis admission +  implicit sepsis admissions

**** p-value was calculated using chi-square for difference in the categorical variables in explicit vs implicit sepsis.

N = number of sepsis admissions

SD = Standard Deviation

The mean age was lowest for explicit admissions at 68.1 years, compared to 72.5 years for implicit admissions. We found that the number of admissions for both explicit and implicit sepsis increased with age. While 4% and 2% of the explicit and implicit admissions were in the 18 to 29 age group, 28% and 36% of the admissions for explicit and implicit sepsis were in patients over 80 years old.

### Number and percentages of ICD sepsis diagnosis codes per diagnosis fields.

In total there were 105,499 ICD-10 codes for explicit sepsis, and 270,346 codes for infection in combination with 240,789 codes for acute organ failure (Table 2).

**Table 2:** Number of ICD-10 sepsis codes in main and 19 secondary diagnosis fields.

Diagnosis field	ICD-10 category		
	Explicit^*^ n^1^(%)	Implicit^**^	
**Diagnosis field (DX)**		**Infection** **n** ^1^ **(%)**	**Acute organ failure n** ^1^ **(%)**
DX 1	58284 (55)	100 078 (37)	24071 (10)
DX 2	24600 (23)	46657 (17)	38170 (16)
DX 3	7158 (7)	32091 (12)	48497 (20)
DX 4	4240 (4)	24800 (9)	39267 (16)
DX 5	3052 (3)	19076 (7)	29110 (12)
DX 6	2408 (2)	14180 (5)	20229 (8)
DX 7	1765 (2)	10198 (4)	13654 (6)
DX 8	1207 (1)	7382 (3)	9241(4)
DX 9	765 (1)	4429 (2)	5648 (2)
DX 10	522 (1)	3025 (1)	3806 (2)
DX 11	409 (<1)	2202(1))	2605 (1)
DX 12	298 (<1)	1643 (1)	1872 (1)
DX 13	201(<1)	1201 (<1)	1292 (1)
DX 14	141(<1)	882 (<1)	968 (<1)
DX 15	131(<1)	707 (<1)	697 (<1)
DX 16	99 (<1)	515 (<1)	514 (<1)
DX 17	75 (<1)	409 (<1)	370 (<1)
DX 18	55 (<1)	359 (<1)	315 (<1)
DX 19	48 (<1)	263 (<1)	254 (<1)
DX 20	41 (<1)	249 (<1)	209(<1)
Total	105 499	270 346	240 789

^1^n = number

* Explicit sepsis codes are retrieved from all admissions (2008-2021) and a discharge with explicit sepsis can include infection and/or codes for acute organ failure at the same admission.

** Implicit codes are retrieved from all admissions (2008-2021) and can include codes for explicit sepsis at the same admission.

**Fig 2:**
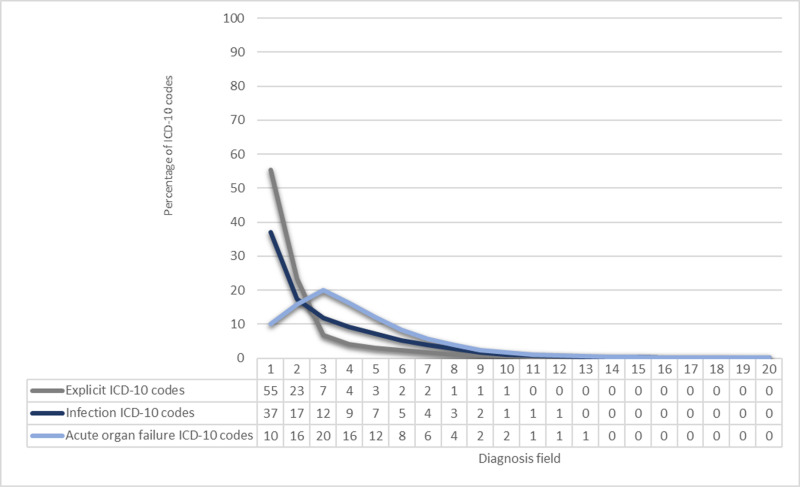
Percentage of ICD-10 codes in diagnoses field one through 20 for explicit sepsis, infection and acute organ failure sepsis codes.

55% of the explicit codes was recorded in the primary diagnosis field, while the same applied for 37% of the infection codes, and 10% of the acute organ failure codes. The proportion of explicit and infection codes peaked in the primary diagnosis field, while for acute organ failure codes this was true in the third diagnosis field (Fig 2).

### Cumulative percentage of sepsis diagnosis.

**Fig 3:**
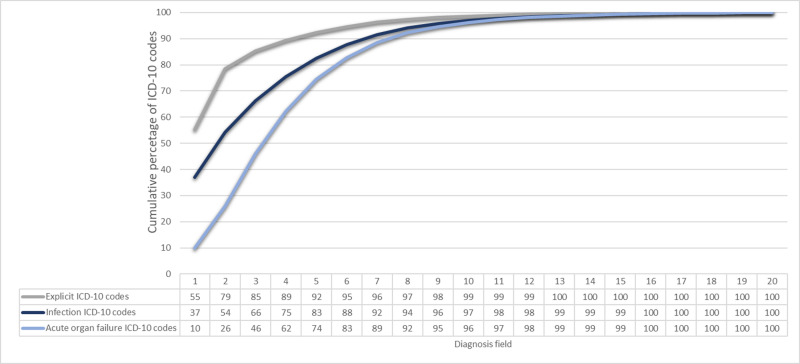
The cumulative percentage of ICD-10 codes for explicit sepsis, infection and organ failure sepsis codes by diagnosis field 1 through 20.

The cumulative percentage reflects the total proportion of cases that have been coded within the designated fields, highlighting the robustness of the coding system in identifying sepsis-related conditions, including infections and acute organ failures. In our study, the cumulative proportion reached 99% in diagnosis field 10 for explicit sepsis codes, and 99% in diagnosis field 13 for implicit sepsis codes, including infection and acute organ failure codes. (Fig 3).

## Discussion

In this nationwide study, we present proportions of explicit and implicit codes as primary condition and up to 19 secondary diagnoses. Our findings reveal that the majority of explicit codes (55%) was listed as the primary diagnosis, while this was true for only 37% of the infection codes and for 10% of the acute organ failure codes. Notably, the cumulative proportion reached 99% in diagnosis field 10 and 13 for explicit and implicit codes, respectively.

To our knowledge, no previous study has examined the necessary medical diagnosis fields to extract implicit sepsis. Our findings on how many diagnosis fields required to capture explicit sepsis are somewhat lower than a previous study commissioned by the World Health Organization, which investigated postoperative explicit sepsis codes among twenty countries, suggesting that at least 15 secondary diagnosis fields are optimal for relevant clinical information using ICD-10 codes [6]. Unlike postoperative sepsis, it is probable that a non-postoperative sepsis discharges (e.g., acute sepsis) will be classified as a primary or at least as an early secondary diagnosis. Therefore, our wider inclusion of specific sepsis codes may account for the differing outcomes.

Information about the number of diagnoses fields used during extraction of data is missing in reporting guidelines for observational studies [13]. One of the challenges when comparing studies is the differences in national ICD-10 coding guidelines. A prior sepsis ICD coding validation study of 22 international studies on a population level compared five strategies [14]. They found that R-codes and explicit sepsis coding strategies may underestimate sepsis incidence by 3.5-fold and 3-fold, respectively. However, in many of these epidemiological studies of sepsis, information about the technical extraction strategy involving the number of diagnosis fields is missing, making it difficult to compare national sepsis incidence. Our study has revealed that extracting sepsis codes in fewer than 10 diagnosis fields for explicit and 13 fields for implicit sepsis may introduce a truncation bias, potentially leading to underestimation of incidence.

### Strengths and limitations

Our study boasts several notable strengths as well as some limitations. Firstly, it draws on data from all public hospitals in Norway spanning 14 years. Secondly, a German study showed that using explicit sepsis had a 59.6% risk of underestimating sepsis, while implicit sepsis had a 2.7% risk of overestimating sepsis. Our approach that cover both implicit and explicit sepsis codes, thus adds to the robustness of our findings, however, it might still be an underestimation of sepsis. Thirdly, as we used the same extraction strategy for sepsis identification previously used by other researchers further strengthens the integrity of our study [4,5,15]. Fourthly, register research is however prone to coding errors, missing diagnostic codes or inconsistencies in the reporting of diagnoses, which could impact the accuracy of the data. In Norway, the efforts to minimize these errors includes mandatory reporting of ICD-10 codes to NPR, and quality checks conducted by the National Service of Validation and completeness analysis. This ensures that our extraction of ICD-10 codes has minimal missing, incomplete, or unknown discharge codes [9]. Lastly, in contrast to many other countries, available numbers of secondary diagnosis fields in the data set to capture events are unlimited in Norway [6]. However, in our study we extracted ICD-10 codes from 19 secondary diagnosis fields due to data minimization. Therefore, we cannot rule out that extraction from more diagnosis fields could have increased the diagnosis fields needed to capture sepsis.

### Implications

Our findings have several important implications for clinical practice and health policy. The fact that the identification of sepsis needs a comprehensive number of diagnostic fields highlights the need to report the number of fields used to extract the codes, and not just which diagnostic codes that are extracted. Only by doing this, the truncation bias can be visible. The differences in the number of diagnostic fields required to capture explicit and implicit sepsis suggest that research guidelines should state this to reduce variability and improve comparability across studies and countries. In order to compare sepsis incidence across studies, future research should report the number of diagnosis fields used.

While our study centers on Norway, it holds significance on an international scale, especially for epidemiological research. We believe that countries and healthcare systems with a limited number of diagnosis fields could greatly enhance their ICD-10 reporting by expanding these fields. Such improvements would not only foster better international and intra-national data comparability but also support epidemiological research, health services analysis, utilization studies, and assessments of care quality.

## Conclusion

In conclusion, our research displays the need for multiple diagnosis fields to accurately capture sepsis data in administrative records, suggesting at least 10 diagnosis fields for explicit sepsis and 13 for implicit sepsis to capture 99% of the cases. The significance of our findings lies in their potential to improve comparability in sepsis research, ultimately benefiting clinical practices and patient care. Future research should focus on validating these findings in different healthcare settings and exploring the impact of coding guidelines on the accuracy of sepsis incidence reporting.
